# Language Outcomes in Deaf or Hard of Hearing Teenagers Who Are Spoken Language Users: Effects of Universal Newborn Hearing Screening and Early Confirmation

**DOI:** 10.1097/AUD.0000000000000434

**Published:** 2017-08-25

**Authors:** Hannah Pimperton, Jana Kreppner, Merle Mahon, Jim Stevenson, Emmanouela Terlektsi, Sarah Worsfold, Ho Ming Yuen, Colin R. Kennedy

**Affiliations:** 1 Faculty of Medicine, University of Southampton, Southampton, United Kingdom; 2 Institute of Cognitive Neuroscience, University College London, London, United Kingdom; 3 Faculty of Social and Human Sciences, University of Southampton, Southampton, United Kingdom; 4 Developmental Science Research Department, University College London, London, United Kingdom; 5 Department of Psychology, Oxford Brookes University, Oxford, United Kingdom; and 6 University of Southampton and University Hospital Southampton National Health Service Foundation Trust, Southampton, United Kingdom.

**Keywords:** Deaf, Early confirmation, Language, Hard of Hearing, Newborn hearing screening, Permanent childhood hearing loss

## Abstract

**Objectives:**

This study aimed to examine whether (a) exposure to universal newborn hearing screening (UNHS) and b) early confirmation of hearing loss were associated with benefits to expressive and receptive language outcomes in the teenage years for a cohort of spoken language users. It also aimed to determine whether either of these two variables was associated with benefits to relative language gain from middle childhood to adolescence within this cohort.

**Design:**

The participants were drawn from a prospective cohort study of a population sample of children with bilateral permanent childhood hearing loss, who varied in their exposure to UNHS and who had previously had their language skills assessed at 6–10 years. Sixty deaf or hard of hearing teenagers who were spoken language users and a comparison group of 38 teenagers with normal hearing completed standardized measures of their receptive and expressive language ability at 13–19 years.

**Results:**

Teenagers exposed to UNHS did not show significantly better expressive (adjusted mean difference, 0.40; 95% confidence interval [CI], −0.26 to 1.05; d = 0.32) or receptive (adjusted mean difference, 0.68; 95% CI, −0.56 to 1.93; d = 0.28) language skills than those who were not. Those who had their hearing loss confirmed by 9 months of age did not show significantly better expressive (adjusted mean difference, 0.43; 95% CI, −0.20 to 1.05; d = 0.35) or receptive (adjusted mean difference, 0.95; 95% CI, −0.22 to 2.11; d = 0.42) language skills than those who had it confirmed later. In all cases, effect sizes were of small size and in favor of those exposed to UNHS or confirmed by 9 months. Subgroup analysis indicated larger beneficial effects of early confirmation for those deaf or hard of hearing teenagers without cochlear implants (N = 48; 80% of the sample), and these benefits were significant in the case of receptive language outcomes (adjusted mean difference, 1.55; 95% CI, 0.38 to 2.71; d = 0.78). Exposure to UNHS did not account for significant unique variance in any of the three language scores at 13–19 years beyond that accounted for by existing language scores at 6–10 years. Early confirmation accounted for significant unique variance in the expressive language information score at 13–19 years after adjusting for the corresponding score at 6–10 years (R^2^ change = 0.08, *p* = 0.03).

**Conclusions:**

This study found that while adolescent language scores were higher for deaf or hard of hearing teenagers exposed to UNHS and those who had their hearing loss confirmed by 9 months, these group differences were not significant within the whole sample. There was some evidence of a beneficial effect of early confirmation of hearing loss on relative expressive language gain from childhood to adolescence. Further examination of the effect of these variables on adolescent language outcomes in other cohorts would be valuable.

## INTRODUCTION

Approximately 1 in 1000 babies is born with bilateral permanent childhood hearing loss (PCHL) of at least moderate severity (>40 dB HL) (Davis et al. 1997). The impoverished access to spoken language that is a consequence of childhood hearing loss places many deaf or hard of hearing (D/HH) children at significant risk of delayed language development (Eisenberg 2007; Moeller et al. 2007; Luckner & Cooke 2010; Moeller & Tomblin 2015). Early identification of D/HH children enables them to receive early intervention to improve the quality of their language input during a “sensitive period” for language development at the beginning of life (Thomas & Johnson 2008; Lyness et al. 2013). However, historically, identification of children with congenital PCHL has been delayed, resulting in many months or years of restricted access to spoken language before identification and intervention (Davis et al. 1997). The advent of universal newborn hearing screening (UNHS) created the opportunity to identify children born with PCHL within the first few days of life, including those children with no known risk factors for the condition. This, in turn, made it possible for these children to be fitted with hearing devices that facilitate access to spoken language (e.g., hearing aids or cochlear implants [CIs]) very early in life and for their families to enroll in early intervention programs to support their child’s developing speech, language, and communication skills (Meinzen-Derr et al. 2011; Kasai et al. 2012; Moeller et al., 2013).

A significant body of evidence from around the world has demonstrated the efficacy of UNHS in increasing rates of early identification of babies born with PCHL (see Thompson et al. 2001; Nelson et al. 2008 for reviews). This includes evidence from a controlled trial undertaken in the Wessex region of England (Kennedy et al. 1998, 2005). The Wessex trial was unique in that UNHS was given/not given according to a controlled experimental regimen, a situation made ethically possible because the screening tests involved were at the time novel and unproven. This created two cohorts of babies that were very similar in all respects other than their exposure to UNHS. In the cohort of babies who were exposed to UNHS, 74% of all cases of true PCHL were referred to audiological services before they were 6 months old, more than double the 31% referred before 6 months in the cohort who had not been exposed to UNHS (Kennedy et al. 2005). A recent population-based study in Australia also reported that UNHS was associated with a reduction in the mean age at which infants with PCHL were identified from 16.2 months to 8.1 months when compared with the contemporary birth cohort in another Australian state that adopted a policy of screening only infants known to be at increased risk of PCHL (Wake et al. 2016).

To determine whether UNHS, and the resulting early identification of PCHL, is associated with the predicted benefits to language outcomes, studies have compared these outcomes between groups of D/HH children who were exposed and not exposed to a program of UNHS and between groups of early- and late-identified children (see Thompson et al. 2001; Nelson et al. 2008; Pimperton & Kennedy 2012 for reviews). Of these studies, three major population-based studies have prospectively examined the effect of exposure (or not) to a UNHS program at birth on subsequent language outcomes (Kennedy et al. 2006; Korver et al. 2010; Wake et al. 2016). D/HH children who were involved in the controlled Wessex trial of UNHS participated in a follow-up study at the age of 6–10 years alongside an additional cohort of D/HH children from Greater London who also varied in their exposure to UNHS (Kennedy et al. 2006). Compared to those not exposed, children in populations exposed to a program of UNHS at birth showed significantly superior receptive language skills but no significant advantages for their expressive language or speech skills. Within the same cohort, confirmation of PCHL at ≤9 months was associated with significant benefits to both receptive and expressive language but not to speech skills. Furthermore, the effect sizes for the early versus late confirmation expressive and receptive language comparisons were larger than those for the UNHS versus no UNHS comparisons. This pattern of findings may be explained by the fact that some babies born in periods with UNHS were not confirmed early and some born in period without UNHS were confirmed early, both of which could account for greater benefits of early confirmation (Kennedy et al. 2006; Pimperton & Kennedy 2012). It is important to note that the benefits associated with early confirmation did not bring the average performance level of these early-confirmed children to the same level as their peers with normal hearing (NH): the D/HH children who were early confirmed still showed significant deficits in both their receptive (1.76 SD below the hearing mean) and expressive (0.59 SD below the hearing mean) language skills. Even with early confirmation and intervention (e.g., provision of hearing aids or CIs), it is likely that D/HH children continue to experience a greater degree of inconsistent access to linguistic input and, hence, accrue reduced cumulative linguistic experience relative to their hearing peers (Moeller & Tomblin 2015), and it is likely that this contributes to their persistent language delays.

Korver et al. (2010) compared language outcomes for 3- to 5-year-old D/HH children who were born in regions of the Netherlands where UNHS was in place with those of D/HH children who were born in regions where there was no UNHS program. They found that the children born in regions where there was no UNHS produced significantly more signed words than the children born in regions with UNHS. The number of signed words used was inversely related to the number of spoken words meaning that the children exposed to UNHS showed an advantage in terms of number of spoken words used. This advantage was not statistically significant, but the authors argued it was clinically important. There were no differences between the UNHS and no UNHS groups in terms of their mean length of utterance or the complexity of the sentences they produced.

Most recently, Wake et al. (2016) looked at outcomes for three populations of children with congenital PCHL: one exposed to UNHS, one contemporary birth cohort exposed to risk factor screening, and one earlier birth cohort exposed to opportunistic detection (i.e., no systematic UNHS or risk factor screening programs). They found that in children without intellectual disability exposure to UNHS was associated with significant population-level benefits to expressive and receptive language skills compared to exposure to risk factor screening and that population language scores improved incrementally from opportunistic detection to risk factor screening to UNHS.

Other studies have also been conducted to look at the effects of UNHS exposure on language outcomes. Yoshinaga-Itano et al. (2000) found significantly higher receptive and expressive language outcomes for D/HH children aged between 9 months and 6 years who had been born in hospitals offering UNHS compared with those born in hospitals that did not. By contrast, Fitzpatrick et al. (2007) did not find any significant advantages in terms of expressive or receptive language outcomes for children who had been screened as newborns compared with those who had not. They suggested that one of the reasons they may have been unable to detect benefits of early confirmation in their study was the inclusion of a relatively high proportion of children with CIs. They argued that for these children, early confirmation was likely to have less of an impact on language outcomes than the age at which they received their implant, that is, when they achieved “access to effective intervention,” and that age at implantation was similar across the screened and unscreened groups. A recent large-scale study in Australia also found that age at implantation was a significant predictor of language outcomes in children with CIs (Ching et al. 2013).

From 2003 onwards, UNHS has been implemented as national or regional policy in numerous countries around the world, including the United States, where in 2009, an estimated 5073 cases of PCHL were detected by UNHS (Howell et al. 2012). This figure accounted for 43.3% of all detected cases of any of the 29 medical conditions for which newborn screening is recommended in the United States. To date, however, no study has followed up infants involved in trials of UNHS through to the teenage years. As a result, the longer-term effects of UNHS and early identification of children who are born D/HH on language outcomes are as yet unknown. Following up the cohort of teenagers from the Wessex and Greater London birth cohorts described earlier whose language skills at primary school age have been reported previously (Kennedy et al. 2006) provided us with a unique opportunity to test whether exposure to UNHS and early identification of PCHL brings significant benefit to outcomes in the teenage years. We have reported elsewhere on the literacy outcomes for this cohort (Pimperton et al. 2016), including significant benefits of early confirmation of PCHL, but not exposure to UNHS, on reading comprehension, the primary outcome in the teenage phase of this cohort study.

In the present article, we focus on a subset of the cohort who were spoken language users and, therefore, able to provide data to address the question of whether exposure to UNHS and confirmation of PCHL by ≤9 months brings benefits to spoken receptive and expressive language outcomes in the teenage years. Consistent with the argument of Fitzpatrick et al. (2007) regarding early confirmation having less of an effect for children with CIs, the benefits of early confirmation to reading comprehension reported in Pimperton et al. (2016) were larger in those D/HH teenagers without CIs. We, therefore, examined whether age at confirmation also has differential importance for the spoken language skills of the D/HH teenagers with and without CIs.

Following this sample of D/HH participants from middle childhood through to adolescence also provided us with a unique opportunity to address the question of whether exposure to UNHS and early confirmation were associated with superior spoken language development during this period. To address this, we examined whether UNHS or early confirmation were associated with variation in language outcomes at 13–19 years (i.e., in the current phase of the study) while adjusting for the level of preexisting language skills, as assessed at 6–10 years.

To summarize, this article addressed two key questions:

1. Are (a) UNHS and (b) early confirmation of PCHL associated with benefits to adolescent language outcomes in spoken language users?
2. Are (a) UNHS and (b) early confirmation of PCHL associated with benefits to relative language gain from middle childhood to adolescence in spoken language users?

## MATERIALS AND METHODS

### Participants

**Fig. 1.**
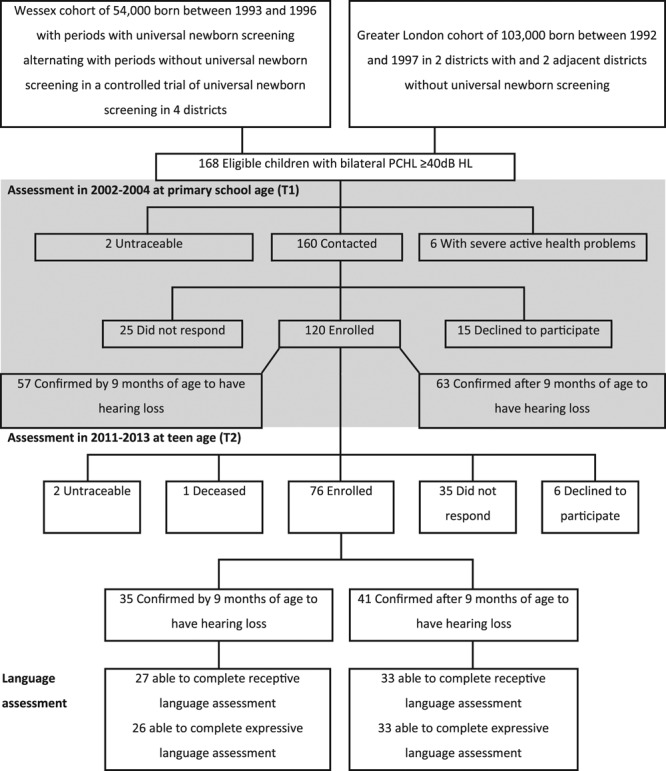
Flowchart of progression of participants through the trial. The grayed section relates to the previous phase of this research study at 6–10 years old (T1), while the section below that relates to the current phase at 13–19 years old (T2). PCHL indicates permanent childhood hearing loss.

The eligible sample for this follow-up study (T2) comprised 120 D/HH teenagers and a comparison group of 63 teenagers with NH who had taken part in the previous phase of this research at primary school at the age of 6–10 years (T1; see Fig. 1). As detailed in Kennedy et al. (2006), those 120 teenagers were drawn from all 160 contactable children with bilateral PCHL of at least 40 dB HL in the better ear identified from a birth cohort of 157,000 children in eight districts of southern England. Children with a known postnatal cause of their hearing loss (e.g., bacterial meningitis) were not included. The children in the sample were born over a 3-year period (1993–1996 inclusive) in four districts in the Wessex region or over a 5-year period (1992–1997 inclusive) in two pairs of adjacent districts in the Greater London region. The four districts in the Wessex subgroup had provided the birth cohort for the Wessex trial, in which a program of universal newborn screening was or was not in place in each pair of districts for birth cohorts born in alternate 4- or 6-month periods. The Greater London subgroup consisted of children born in the only two districts in the United Kingdom that provided UNHS for PCHL in the early 1990s and in two other adjacent districts. Protocols for the identification, confirmation, and management of PCHL were similar at all sites apart from variation in the details of UNHS provision (Tucker & Bhattacharya 1992; Watkin & Baldwin 1999; Kennedy et al. 2005). The children exposed to UNHS and those who were not were, in all but a small number of cases, treated by the same audiological service providers.

Seventy-six of the 120 D/HH teenagers and their families who had been assessed at primary school age agreed to participate in this follow-up phase of the research. Of these 76 D/HH teenagers, 60 and 59 completed the receptive and expressive language assessments, respectively, and were, therefore, included in the present study on spoken language outcomes (see Fig. 1). Those who did not complete the assessments either used British Sign Language as their preferred language, rendering these spoken English assessments inappropriate, or had severe additional disabilities that precluded the development of sufficient language to attempt the tests.

The eligible comparison group of 63 teenagers with NH who participated at T1 was drawn from the same birth cohorts as the group of 120 D/HH children. Thirty-eight of the 63 (60%) teenagers with NH who had participated at T1 took part in the present study (see Fig. 1). All 38 hearing teenagers completed both the receptive and expressive language assessments.

### Procedure

This study was approved by the Southampton and South West Hampshire Research Ethics Committee. Written informed consent for participation in the study was obtained from principal caregivers and from the teenage participants. Each teenage participant was assessed by a trained researcher who was unaware of their audiological history. Testing was undertaken in a quiet room at the teenager’s home or at their school according to their expressed preference.

### Spoken Language Skills Were Assessed With the Following Measures

#### Receptive Language

The Test for Reception of Grammar Version 2 (TROG-2; Bishop 2003), standardized on the age range 3 to 16 years 11 months, as well as with adults, was used to assess participants’ receptive skills for spoken English grammar. Items in the task assess understanding of increasingly complex grammatical contrasts, including plurals, passives, negatives, and relative clauses. Participants must point to a picture from a choice of four alternatives that corresponds to a spoken sentence.

The British Picture Vocabulary Scale Third Edition (BPVS-3; Dunn & Dunn 2009), standardized on the age range 3 years to 16 years 11 months, provided a measure of the participants’ receptive skills for spoken English vocabulary. Participants must point to a picture from a choice of four alternatives that corresponds to a spoken word. Earlier editions of both the BPVS and the TROG were used to measure the participants’ receptive language skills at primary school age (T1).

#### Expressive Language

The Expression, Reception and Recall of Narrative Instrument (ERRNI; Bishop 2004), standardized on the age range 4 years to adults, provided a measure of participants’ expressive spoken language skills. Participants were required to produce a narrative based on a series of picture cues and subsequently to reproduce that narrative without the support of the pictures. Their narrative productions were audio-recorded, subsequently transcribed, and scored according to the ERRNI manual to produce three scores: an initial score for the quality of their initial narrative, a recall score for the quality of their recalled narrative, and a mean length of utterance (MLU) score, which reflected the average length of their utterances in words across both the initial and recall narratives. An inter-rater reliability exercise, following Whitehouse et al. (2009), was carried out to check the reliability of the scoring: 12 randomly selected narratives (12% of the total) were transcribed and scored by a second rater. There was good agreement (intraclass correlations, *r*_ic_) between the two ratings for all three scores (initial, *r*_ic_ = 0.82; recall, *r*_ic_ = 0.90; MLU, *r*_ic_ = 0.95).

The measure used to assess expressive language skills at primary school age (T1), the Renfrew Bus Story Test (Renfrew 1995), was designed for use with 3–8 year olds. This test involved children listening to a story told by the experimenter while viewing a series of pictures that corresponded to the story. They then had to tell the story in their own words (i.e., produce their own narrative), using the pictures as prompts. Two scores were derived from this measure, reflecting both the inclusion of relevant information in the narrative and the length of utterances produced, and were combined into an expressive language composite score (Kennedy et al. 2006). The ERRNI was selected for this current phase of the study because it was similar in design to the Bus Story Test, enabling the derivation of both a score for the information content of the narrative produced, as well as a measure of utterance length, and had been designed for an age range within which our participants fell.

#### Nonverbal Ability

A 20-minute timed version (Hamel & Schmittman 2006) of Raven’s Standard Progressive Matrices Plus (Raven’s SPM+; Styles et al. 1998) was used to assess nonverbal ability. Participants were given 20 minutes to work their way through a series of progressively more difficult matrix reasoning puzzles. Raw scores reflecting the total number of correct items out of a possible 60 were calculated.

#### Demographic and Audiological Characteristics

Other characteristics of the teenager and their family, including maternal education level and languages used in the home, were also documented. The most recently available audiological data were documented from audiology and CI center records. Severity of hearing loss was categorized from the most recent audiological records as moderate (40–69 dB HL), severe (70–94 dB HL), or profound (≥95 dB HL) according to four-frequency averaging of the pure-tone thresholds from 500 to 4000 Hz for the better ear. For participants with CIs, we collected unaided pure-tone thresholds obtained during assessment for implantation.

### Analysis Strategies

#### Effects of UNHS and Early Confirmation

For the purpose of comparisons within the group of D/HH teenagers, we used norms obtained from the participating children with NH (Kennedy et al. 2006). The group mean score and standard deviation score for a particular measure in teenagers with NH were used to derive age-adjusted *z* scores for the D/HH teenagers on that measure. When language outcomes were examined in this cohort at 6–10 years, the BPVS and TROG *z* scores were averaged into a receptive language composite, and the information and sentence length scores from the ERRNI were averaged into an expressive language composite (Kennedy et al., 2006). To check the validity of using the same composite structure at the current time point, correlations between the measures were examined and a principal component analysis was conducted. The two receptive language measures (BPVS and TROG) showed strong positive correlations (n = 98, r = 0.71, *p* < 0.001), and in the principal component analysis, both loaded highly (0.92) on the first component. They were, therefore, combined into a single receptive language composite for analysis purposes. Of the three expressive language scores, the two information scores (initial and recall) showed strong positive correlations with each other (n = 97, r = 0.71, *p* < 0.001) but weaker relationships with the MLU score (n = 97, r = 0.20, *p* = 0.048 and n = 97, r = 0.26, *p* = 0.009, respectively). A principal component analysis on these expressive score identified a single component with an eigenvalue greater than 1. On this component, the two information scores had loadings greater than 0.5 (initial 0.88 and recall 0.90). The MLU had a loading of 0.49. The two information scores were, therefore, combined into an expressive information composite, and the MLU score was reported separately for analysis purposes. Thus, the three language outcomes examined in this study were a receptive language composite (BPVS and TROG scores), an expressive information composite (ERRNI initial and recall information scores), and expressive MLU score.

We prespecified the definition of “early” confirmation of PCHL as confirmation at ≤9 months of age, consistent with the definition used in our previous trial of UNHS (Kennedy et al. 1998) and our evaluation of language at primary school age (Kennedy et al. 2006). We separately assessed the associations between (a) exposure to UNHS (i.e., birth during periods when UNHS was in place) and (b) confirmation of PCHL at ≤9 months of age and each of the three receptive and expressive language scores (receptive, expressive information, expressive MLU) before and after adjustment in a multiple linear regression for severity of hearing loss, maternal education, and nonverbal ability, which were prespecified as potential confounders of the study outcomes (Kennedy et al. 2006), and English as an additional language in the home, which was identified as a potential confounder of the outcomes at the current time point because of unequal distribution between the early and late confirmed groups (Pimperton et al. 2016). We tested for an interaction between the effects of (a) UNHS versus no UNHS and (b) early versus late confirmation of PCHL and cochlear implantation (CI versus no CI) by entering an additional term reflecting this interaction into regression analyses, predicting the combined receptive and expressive language scores.

Normality and homogeneity of the residual variance were examined for all measures to ensure that the regression models were appropriate. All reported *p* values are 2-sided, and 95% confidence intervals (95% CIs) are given.

#### Language Development From Childhood to Adolescence

To examine whether (a) exposure to UNHS and (b) early age at confirmation were associated with variability in relative language gains from middle childhood to adolescence, a hierarchical multiple regression analysis was conducted, which assessed whether UNHS exposure or age at confirmation predicted significant unique variance in T2 language (assessed at the current assessment time point, aged 13–19 years) when adjusted for T1 language (assessed at the previous assessment time point, aged 6–10 years), as well as the confounding variables (severity of hearing loss, maternal education level, nonverbal ability, and English as a main language at home). The analysis was run separately for receptive and the two expressive language measures. Normality and homogeneity of the residual variance were examined for all measures to ensure that the regression models were appropriate. This analysis necessarily included only D/HH participants who had provided spoken language data at both time points (receptive, N = 59; expressive, N = 54). For this longitudinal element of the analysis, it was important to directly compare the same D/HH participants relative to the same NH control group at both time points (T1 and T2). We, therefore, recalculated the T1 language *z* scores for the D/HH participants using the norms only from those participants with NH who provided the norms for the T2 phase of the research (N = 38) and used these in the analysis.

## RESULTS

### Participants

Table 1 presents descriptive statistics on key Time 1 demographic variables for the teenagers who did and did not provide spoken language data for the present study. The D/HH teenagers who provided spoken language data in the present study did not differ significantly from those who did not provide spoken language data in terms of age, sex, severity of hearing loss, use of a CI, birth in periods with UNHS, confirmation at ≤9 months, or maternal education level (all *p’*s >0.10), but there was a nonsignificant tendency for those who did provide spoken language data to be more likely to have English as the main language at home [χ^2^ (2, N = 98) = 5.22, *p* = 0.07]. The teenagers with NH who provided data in the present study did not differ significantly from those who were lost to follow-up in terms of age, sex, and use of English as a first language at home (all *p*’s >0.10) but compared to those who were lost, those who were retained showed higher maternal education levels [χ^2^ (2, N = 98) = 6.13, *p* = 0.05]. Both the D/HH and NH groups who provided T2 spoken language data showed higher receptive language *z* scores at T1 than their counterparts who did not [D/HH: *t* (99) = 1.98, *p* = 0.05; NH: *t* (61) = 2.22, *p* = 0.03].

**TABLE 1.**
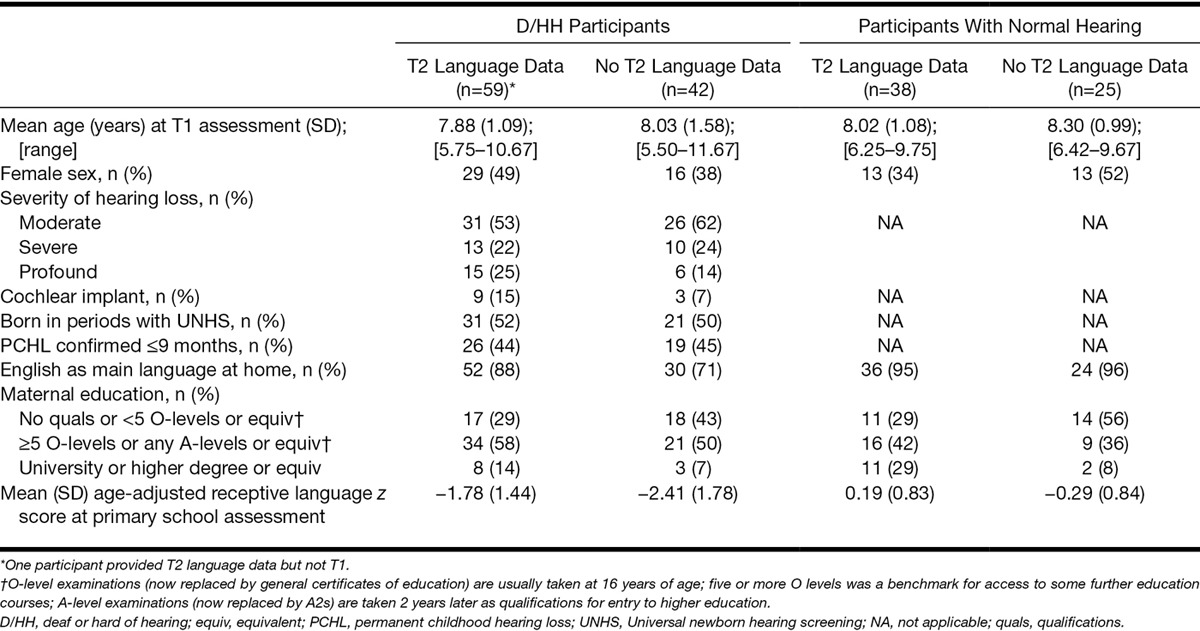
Demographic characteristics at T1 (6–10 years assessment time point) for participants who provided spoken language data at T1 and who either did or did not provide spoken language data in the present study of language outcomes in teenagers (T2)

Table 2 presents descriptive statistics on key Time 2 demographic variables for the teenagers who provided language data for this study. The D/HH teenagers did not differ significantly from the teenagers with NH with respect to gender, nonverbal ability, or use of English as the main language at home (all *p*’s >0.10). The teenagers with NH were significantly younger than the D/HH teenagers [*t* (96) = 2.65, *p* = 0.01], and there was a nonsignificant tendency for them to have higher maternal education levels [χ^2^ (2, N = 98) = 5.22, *p* = 0.07]. Scores were age-adjusted before analysis, and maternal education was adjusted for in the group comparisons.

**TABLE 2.**
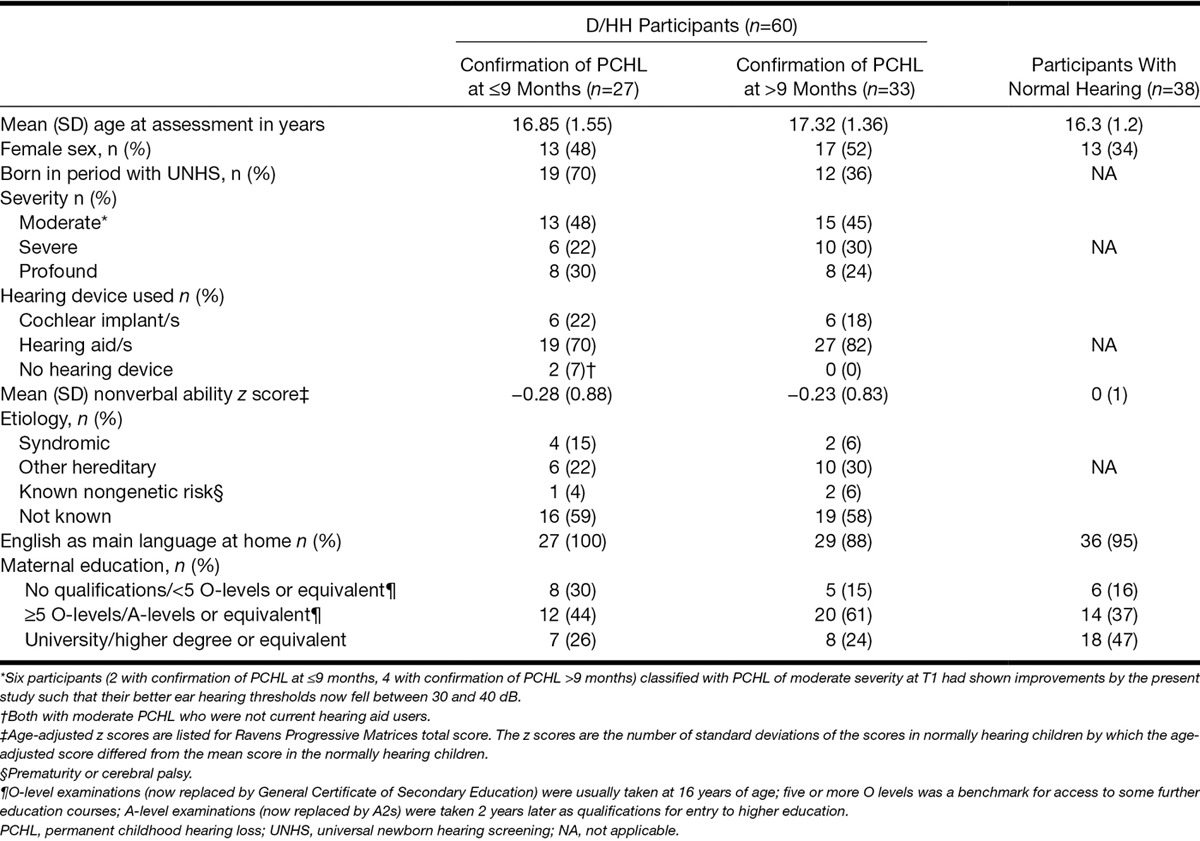
Characteristics of participating teenagers who provided spoken language data

The D/HH participants confirmed at ≤9 months did not differ significantly from those who were confirmed at >9 months with respect to age, gender, severity of hearing loss, use of a CI, nonverbal ability, etiology, and maternal education level (all *p*’s > 0.10; Table 2). There was a nonsignificant tendency for more teenagers confirmed at ≤9 months to have English as the main language at home [χ^2^ (1, N = 60) = 3.51, *p* = 0.06]. This variable was adjusted for in the group comparisons. Those exposed to UNHS did not differ significantly from those not exposed to UNHS with respect to age, gender, severity of hearing loss, use of a CI, nonverbal ability, etiology (all *p*’s >0.10). There was a nonsignificant tendency for lower maternal education in the group exposed to UNHS [χ^2^ (2, N = 60) = 5.34, *p* = 0.07]. This variable was adjusted for in the group comparisons.

### Language Outcomes NH Versus D/HH

The teenagers with NH showed significantly higher adjusted mean receptive language *z* scores than the D/HH teenagers, but no significant advantage in terms of expressive language *z* scores (Table 3). This contrasts with the findings in this cohort aged 6–10 years, when the D/HH participants showed significant deficits in both receptive *and* expressive language relative to the comparison group with NH (Kennedy et al. 2006). When we looked only at those participants who provided receptive and expressive language data at both time points to make a direct comparison, the same pattern of results was evident in that while the magnitude of the receptive language deficit shown by the D/HH participants (N = 54) relative to the participants with NH (N = 38) remained similar from T1 to T2 (T1 *M* Difference = −2.01, 95% CI = −2.50 to −1.51; T2 *M* Difference = −1.78, 95% CI = −2.45 to −1.10), the expressive deficits for both MLU (T1 *M* Difference = −0.96, 95% CI = −1.52 to −0.39; T2 *M* Difference = −0.15, 95% CI = −0.55 to 0.25) and information score (T1 *M* Difference = −1.28, 95% CI = −1.85 to −0.71; T2 *M* Difference = −0.20, 95% CI = −0.64 to 0.24) were much reduced.

**TABLE 3.**
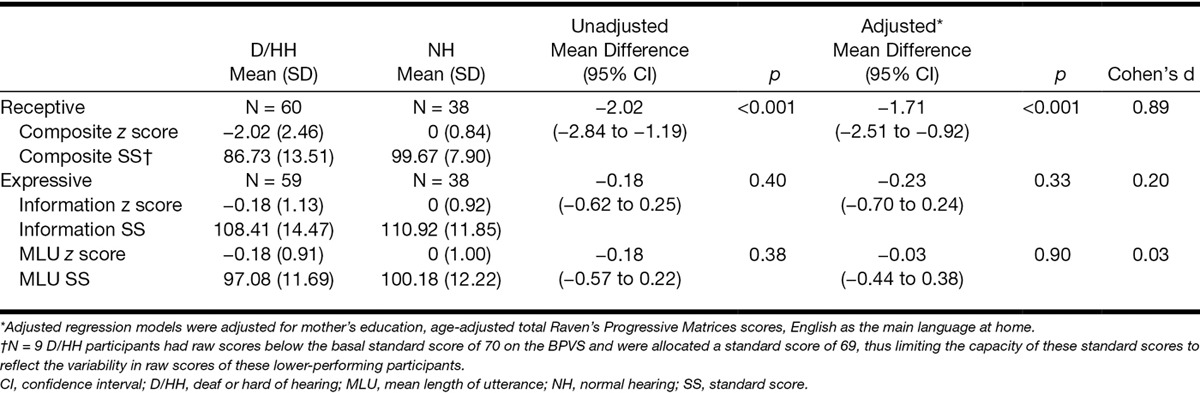
Group mean receptive and expressive language z scores and standard scores for D/HH teenagers and teenagers with normal hearing

To examine whether the patterns of language deficits shown by the D/HH group in this study were a function of the NH reference group used, we also examined standard scores on the receptive and expressive language measures to assess their performance relative to the larger hearing samples on which the tests were standardized. Standard scores (M = 100, SD = 15) were available for all participants who completed the ERRNI. The D/HH group did not show evidence of substantial deficits on this task when examining their scores relative to the standardization sample; their standard scores were close to or above the mean of the standardization sample and were similar to those obtained by the NH reference group who participated in this study (Table 3).

Standard scores (M = 100, SD = 15) were available for all participants on the TROG. For the BPVS, standard scores (M = 100, SD = 15) were available for participants up to 16;11 years. For all participants in our sample over this age, we allocated them the standard score for the highest available age bracket (16;09–16;11). For participants whose raw score placed them below the basal standard score of 70 (N = 9, all D/HH), we allocated them a score of 69. This limited the capacity of these standard scores to reflect variability in raw scores for these lower scoring participants. The mean receptive standard score (TROG standard score + BPVS standard score/2) for the D/HH group was around 1 SD below the standardization mean of 100, while the mean standard score for the NH group was very close to the standardization mean (Table 3).

### Effects of UNHS

**Fig. 2.**
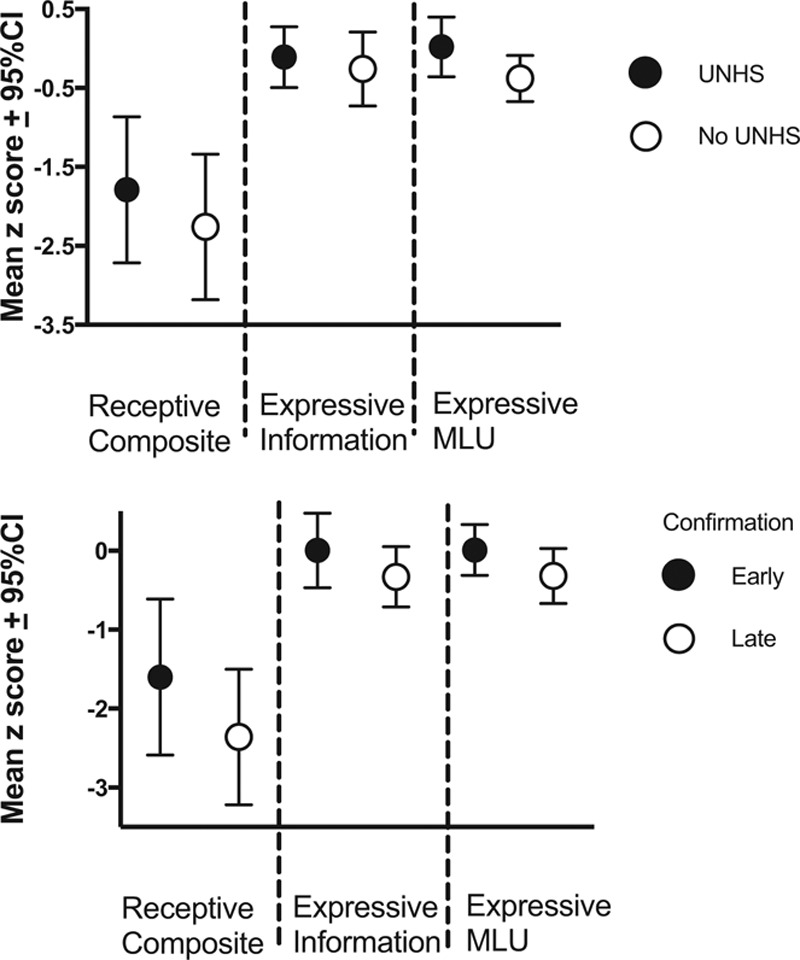
Mean age-adjusted receptive and expressive language *z* scores for the early- (filled circles) and late- (unfilled circles) confirmed and the universal newborn hearing screening (UNHS; filled circles) and no UNHS (unfilled circles) D/HH, deaf or hard of hearing (D/HH) participants. Error bars show 95% confidence intervals around the mean.

Compared to birth during a period without UNHS, birth during a period with UNHS was not associated with significantly higher expressive and receptive language *z* scores (see Table 4 and Fig. 2). Effect sizes were all in the direction of favoring the UNHS group and were of small size.

**TABLE 4.**
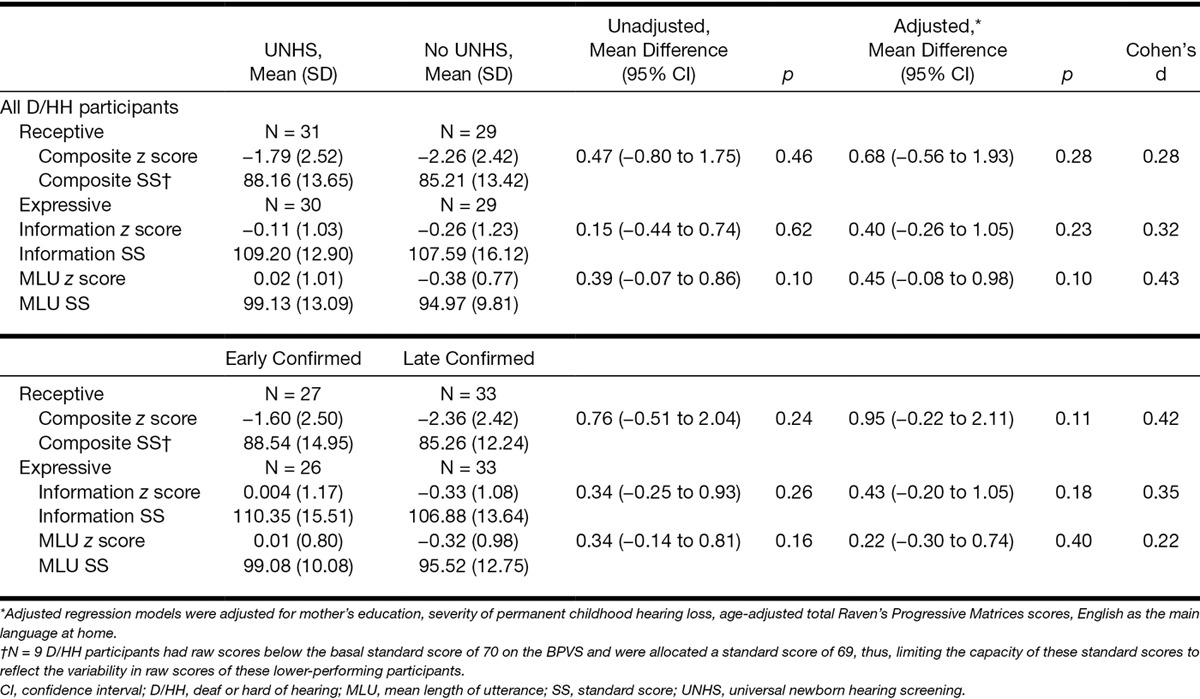
Receptive and expressive language z scores for D/HH teenagers by birth in periods with and without universal newborn hearing screening and by age of confirmation of hearing loss

The additional interaction term reflecting the interaction between the presence of a CI and the effects of UNHS versus no UNHS on overall language was not significant (*p* = 0.22).

### Effects of Early Confirmation

Compared to confirmation of PCHL at a later age, confirmation of PCHL at ≤9 months of age was not associated with significantly higher receptive and expressive language *z* scores for the whole sample (see Table 4 and Fig. 2). Effect sizes for all three language outcome variables were in the direction of favoring the early confirmed group and were of small size.

The additional interaction term reflecting the interaction between the presence of a CI and the effects of early versus late confirmation of PCHL on overall language was significant (*p* = 0.03) suggesting that age at confirmation may be differentially affecting the language outcomes of those D/HH participants without CIs (N = 48) compared to those with CIs (N = 12); therefore, results were also examined separately for the CI versus no CI groups.

For the D/HH participants without CIs (N = 48), confirmation of PCHL at ≤9 months was associated with significantly higher receptive, but not expressive, language scores after adjustment for the effects of severity of hearing loss, maternal education level, nonverbal ability, and the presence of English as an additional language in the home (see Table 5). Effect sizes for all three language outcome variables were in the direction of favoring the early confirmed group and were of medium size.

For the D/HH participants with CIs (N = 12), numbers were too small to carry out parallel regression analyses but descriptive statistics comprising unadjusted means (SDs) and mean differences are reported (Table 5) and indicate lower language scores in all three domains for the early confirmed participants.

### Language Development From Childhood to Adolescence

For all D/HH participants who provided receptive language data at both time points (N = 59), receptive language *z* score at T1 was entered at Step 1 of the hierarchical linear regression analysis predicting receptive language *z* score at T2. Severity of hearing loss, nonverbal ability, maternal education level, and English as a main language at home were entered at Step 2, and finally, exposure to UNHS (in model 1) or age at confirmation (in model 2) was entered as a dichotomous predictor variable at Step 3. In both models, adding group membership (UNHS versus no UNHS in Model 1, early versus late confirmed in Model 2) at Step 3 did not predict significant additional unique variance in T2 receptive language outcomes (see Table 6).

**TABLE 5.**
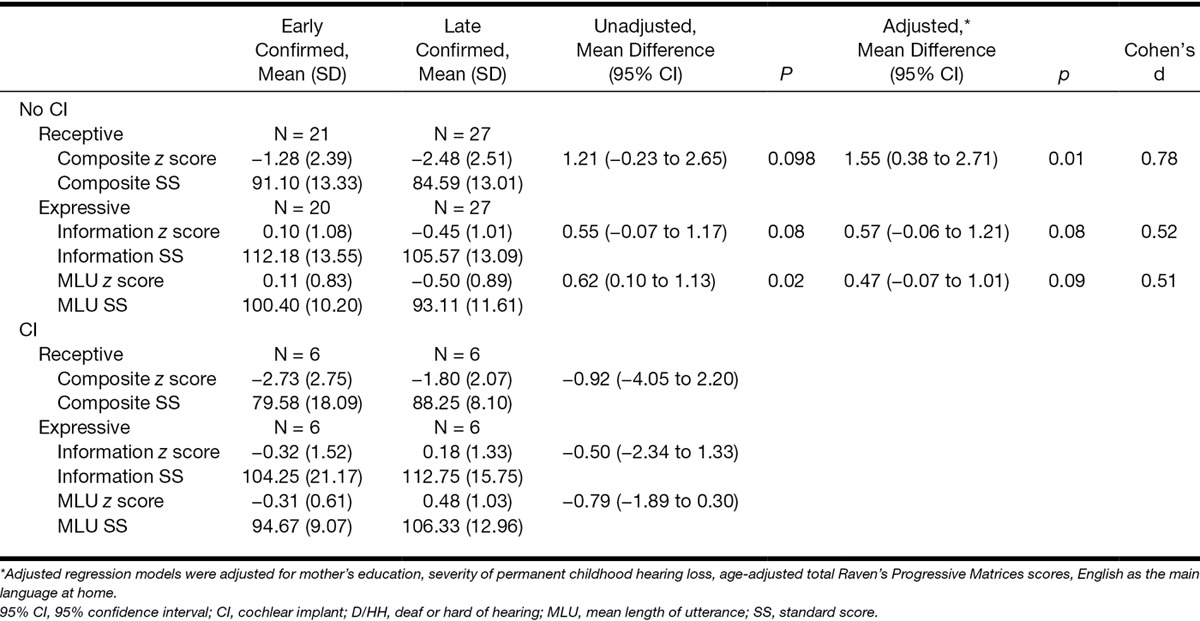
Receptive and expressive language z scores by age of confirmation of hearing loss for D/HH teenagers stratified by cochlear implant vs. no cochlear implant

**TABLE 6.**
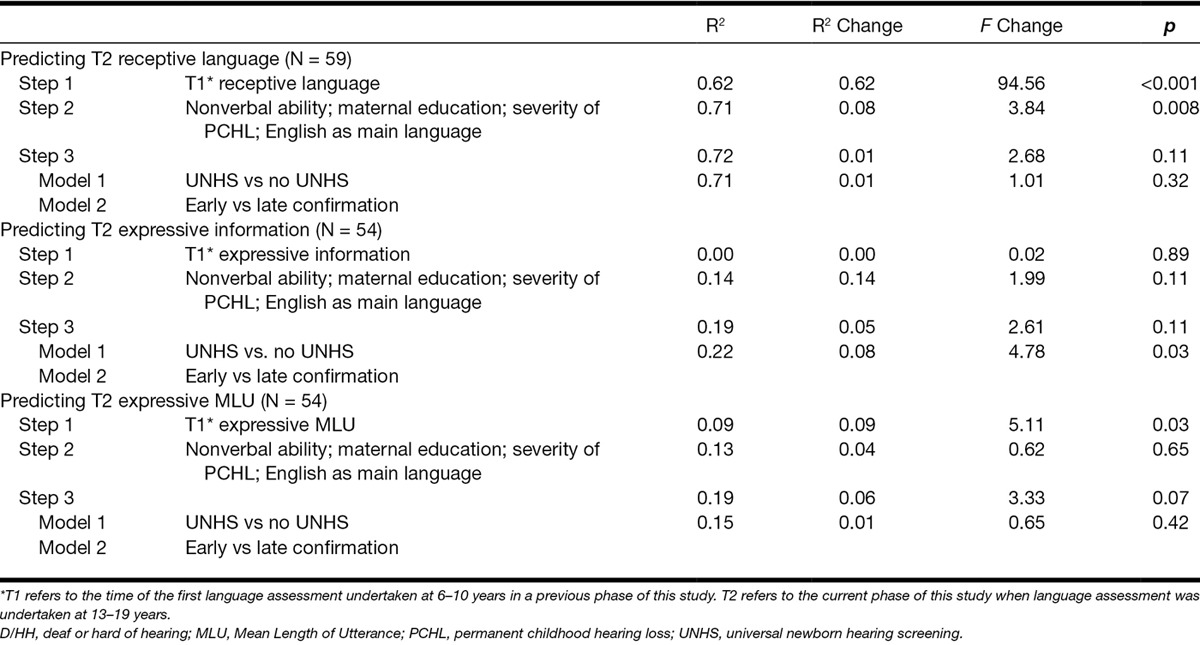
Hierarchical multiple linear regression analyses predicting language scores at T2 for the D/HH participants*

Parallel regression analyses were run predicting the two T2 expressive language outcomes (information and MLU) for all D/HH participants who provided expressive language data at both time points (N = 54), with the equivalent expressive language score from T1 entered at Step 1. The regression models for expressive language accounted for much lower proportions of the variance in T2 expressive language than was the case for receptive language because the relationship between T1 and T2 expressive language scores was much weaker than that between T1 and T2 receptive language scores. Exposure to UNHS entered at Step 3 of the model did not account for significant unique variance in either of the T2 expressive language outcomes. Age at confirmation entered at Step 3 accounted for significant unique variance in expressive information score but not in expressive MLU (see Table 6).

Running the same set of analyses for the D/HH participants without CIs who had provided language data at both time points (receptive N = 48; expressive N = 44) produced the same pattern of significant effects of age at confirmation as in the whole sample, though in all cases, the percentage of unique variance explained by age at confirmation was higher than it was for the whole D/HH group; age at confirmation entered at Step 3 in the model predicted significant unique variance in T2 expressive information [16%; *F*(1, 37) = 8.42, *p* = 0.01] but not T2 expressive MLU [5%; *F*(1, 37) = 2.38, *p* = 0.13) scores or T2 receptive scores (2%; *F*(1, 41) = 3.75, *p* = 0.06). The number of D/HH participants with CIs who had provided language data at both time points (receptive N = 11; expressive N = 10) was too small to run these hierarchical multiple linear regression analyses for that subgroup.

## DISCUSSION

This follow-up study in a cohort of spoken language–using D/HH teenagers found no significant benefits of UNHS or confirmation of PCHL at ≤9 months of age on receptive or expressive language outcomes in adolescence. For all outcomes, UNHS and early confirmation were associated with higher language scores but effect sizes were of small size (range of Cohen’s *d*’s 0.22–0.43), and the differences between groups were not significant. The lack of significant effects of UNHS and early confirmation on teenage language outcomes within the whole sample differs from earlier findings with this cohort in middle childhood (Kennedy et al. 2006) when UNHS exposure was associated with significant benefits to receptive language and early confirmation with significant benefits to both receptive and expressive language. Sample attrition over the approximately 8 years between these two assessment time points, coupled with inclusion only of those participants who were spoken language users, reduced the sample size for the current phase of this study. Additionally, both the screened and unscreened and the early and late confirmed groups of teenagers showed very high within-group variation in their language outcomes. This high within-group variability shown by the D/HH teenagers, in combination with attrition of the sample over time, may have limited the power of the study to detect significant effects of UNHS and early confirmation. The question of benefit of UNHS and early confirmation to spoken language skills should, therefore, be further examined in other population-based cohorts (e.g., Korver et al. 2010; Wake et al. 2016) when they reach adolescence. Individual participant meta-analysis combining data across studies is likely to be valuable and should also be considered.

Subgroup analysis indicated a differential benefit of early confirmation for those in the D/HH sample with and without CIs. Caution must be taken in interpreting the results of subgroup analyses and, particularly, in this case where numbers of participants with CIs were small; however, these results indicate that early confirmation was not bringing the same benefits to the language outcomes of those D/HH teenagers in the study with CIs as it did to those without CI. For the participants in the D/HH sample who did not have a CI (this subgroup comprised 80% of the overall sample), the effects of UNHS and confirmation of PCHL at ≤9 months of age were larger (range of Cohen’s *d*’s 0.46–0.78) than they were for the whole sample, and confirmation of PCHL at ≤9 months of age was associated with significant benefits to receptive language. By contrast, benefits of confirmation of PCHL at ≤9 months of age were not apparent in the small group of participants with CIs. This is consistent with the suggestion of Fitzpatrick et al. (2007) that the age at which these children access effective intervention (i.e. the age they receive their CI) is likely to have more effect on their language outcomes than the age at which their PCHL is identified, with the observation that age at implantation predicts early language outcomes for D/HH children with CIs (Ching et al. 2013) and with our own findings regarding literacy outcomes in this cohort (Pimperton et al. 2016). The participants in this study were born at a time when age at implantation was typically much later than it is in the present day even after early confirmation of PCHL: only one participant in this study received a CI before the age of 3 years.

It is important to recognize, however, that age at implantation is unlikely to be the only variable influencing language outcomes for the participants with CIs: other factors related to preimplant auditory experience are known to be important explanatory variables for variation in language outcomes for children with CIs (Geers et al. 2008; Niparko et al. 2010; Boons et al. 2012). A recent large-scale longitudinal study of children with CIs suggested a pattern of decreasing influence of age at implantation on language outcomes as children move through middle childhood and discussed other factors, such as the amount of time the CI is worn each day, that may drive variation in language outcomes (Dunn et al. 2014). The sample of participants with CIs in the present study was small but actually showed a trend in the opposite direction to the D/HH participants without CIs, with the late-confirmed group showing superior language skills to the early-confirmed group. One possible explanation for this is that some of the participants with CIs in the late-confirmed group may have had an early, undocumented, progressive loss. This would have meant that, first, they may have screened negative on UNHS and been more likely to be late confirmed and, second, that they would have had some time with additional residual hearing before implantation, a factor previously shown to predict better language outcomes after implantation (Geers et al. 2008; Niparko et al. 2010).

As discussed earlier, within the subgroup of D/HH participants without CIs, early confirmation was associated with significant benefits to receptive but not expressive language outcomes in adolescence. A lack of sensitivity of the expressive language measure to the aspects of language that are particularly vulnerable in D/HH children (e.g., inflectional morphology (Tomblin et al., 2015)) may have contributed to this pattern of findings; on the expressive language measure used in this study, MLU was calculated in words not in morphemes, which would make it insensitive to deficits in inflectional morphology, whereas one of the receptive language measures explicitly assessed elements of inflectional morphological knowledge. In keeping with this potential differential sensitivity of the receptive and expressive measures, the receptive language measures indicated significant deficits for the D/HH group relative to the NH group, while the expressive language measure did not, a finding further considered at the end of the discussion. Rescoring the narrative output from the ERRNI to more closely align with the constructs measured by the TROG may have increased the sensitivity of this measure to differences in expressive morphology and syntax between the early- and late-confirmed participants, as we found to be the case with a rescoring of the Bus Story Narratives produced in the earlier phase of the present study when the participants were aged 6–10 years (Worsfold et al. 2010).

As with the influence of age at implantation on language outcomes of D/HH teenagers with CIs, it is important to emphasize that UNHS and early confirmation of PCHL is likely to be just one of a multitude of variables that influence spoken language outcomes for D/HH teenagers without CIs. A recent large-scale, longitudinal study of children with mild to severe hearing loss identified variables associated with individual differences in their language outcomes (Moeller & Tomblin 2015). These variables related to access to language input and included variability in the quality of hearing aid fitting, consistency of use of hearing aids, and characteristics of caregiver language input. It may be the case that these variables associated with individual differences in language outcomes in D/HH preschoolers have cumulative effects by the teenage years.

When examining the effects of UNHS and early confirmation on relative language gain from middle childhood to adolescence, exposure to UNHS did not account for significant unique variance in language scores at 13–19 years (T2) beyond that accounted for by existing language scores at 6–10 years (T1). Early confirmation of PCHL accounted for significant unique variance in T2 expressive information score after adjusting for T1 expressive information score but not for T2 expressive MLU or receptive scores after adjusting for corresponding T1 scores. The same pattern of significant effects of early confirmation was evidenced in the subgroup of participants without CIs; early confirmation of PCHL predicted significant unique variance in T2 expressive information but not in expressive MLU or receptive. These findings suggest that the D/HH teenagers who had their hearing loss confirmed early had made greater relative progress in one element of their expressive language skills over the years subsequent to middle childhood, raising the possibility that earlier exposure to language leading to better language skills in middle childhood may bring lasting benefits to later language development. The relationship between expressive language *z* scores in middle childhood with those in adolescence was much weaker than was the case for receptive language, and consequently, the longitudinal expressive language models were a less good fit to the data; indeed, for expressive information, T1 scores did not account for any variance in T2 scores. This much greater stability in receptive language skills may have contributed to the lack of a significant effect of early confirmation on relative growth in receptive language skills from T1 to T2. The lack of stability for expressive language may also indicate that the T1 and T2 expressive language measures are not necessarily measuring the same sets of skills at both time points, a possibility considered further at the end of the discussion.

Where there were significant benefits detected in this study, those were of early confirmation, not of exposure to UNHS. Not all D/HH children who were exposed to UNHS in this sample were confirmed early, and some of those who were confirmed later *were* exposed to UNHS: 70% of the early-confirmed participants and 36% of the late-confirmed participants in this study had been exposed to UNHS at birth. UNHS is designed to take effect by allowing early confirmation of PCHL and, consequently, early intervention to optimize the child’s early communicative environment. If a child is exposed to a UNHS program but not screened, or is screened but not early confirmed, then they are unable to access early intervention, and the intended benefits of UNHS cannot be realized. This emphasizes the importance of ensuring that effective pathways are in place to follow-up children picked up by UNHS, confirm the presence of PCHL, and initiate intervention within the shortest possible timeframe (Kasai et al. 2012; Moeller et al. 2013). The models that we constructed took account of maternal education, English as first language, and nonverbal ability, but it is still possible that the relatively larger benefits to language associated with early confirmation of PCHL, compared to those associated with birth in periods of UNHS, could have been contributed to by residual confounding between other drivers, such as family engagement and efficacy, of both earlier confirmation and superior language outcomes.

In contrast to the previous phase of this study when the D/HH children aged 6–10 years showed significant deficits relative to the NH group in both receptive and expressive language (Kennedy et al. 2006), the D/HH teenagers showed significant deficits relative to the NH group in receptive, but not expressive, language skills. There was some evidence of selective loss from the study of NH participants whose mothers had lower educational qualifications at the earlier assessment time point. However, when looking at a directly comparable sample (i.e., only those D/HH and NH participants who provided receptive and expressive language data at both time points), the pattern of apparently resolved deficits on the expressive language task in the face of persistent deficits on the receptive language task for the D/HH group was still clear, suggesting that it cannot be attributed to changes in the study sample between the two assessment time points. Additionally, when examining standard scores for the D/HH group, which provide an indication of how they are performing relative to the large hearing samples on which the language tests were standardized, the D/HH showed standard scores that were near or above the mean for expressive language and 1 SD below the mean for receptive language, suggesting that the pattern of D/HH language performance was not a function of the NH comparison group included in this study.

The question remains then as to why the expressive language deficit of the D/HH participants is no longer evident while their receptive language deficit has remained consistent from the primary to the secondary school years. One possibility relates to the tasks used to assess receptive and expressive language. While the receptive language tasks were the same at both assessment time points, the expressive language task used at 6–10 years (the Bus Story; designed for 3–8 year olds) differed from that used at 13–19 years (the ERRNI; designed for use from 4 years of age to adulthood) because the ERRNI is a more age-appropriate assessment for a teenage sample. The ERRNI was, nevertheless, selected as a measure that was as comparable as possible to the Bus Story: both tests involve the participant viewing a series of pictures that tell a story and producing a narrative based on the pictures. They differ, however, in that the Bus Story test administrator gives the children a model spoken narrative, whereas in the ERRNI, they must produce their own narrative solely based on the pictures. Skills related to the reception and retention of the model story in the Bus Story assessment may, therefore, have given the hearing children an advantage at the earlier assessment time point, which was not the case with the ERRNI at the second assessment time point. Indeed, this differential dependence of the receptive and expressive language tasks on auditory access may have been a contributing factor to the discrepancy we observed at the present time point in terms of D/HH deficits relative to the NH group on these tasks. This would be in addition to the factor discussed earlier regarding the differential sensitivity of the receptive and expressive language measures to the aspects of language that are most challenging for D/HH individuals (e.g., inflectional morphology).

It is also possible that some of the D/HH participants have learnt, as they have got older, to use compensatory language strategies that can be successfully deployed on the expressive narrative task, but not on the receptive language tasks where there is simply a right or wrong answer. One strategy, for example, might be to produce a lengthy response to the request for a narrative, which would be more likely to cover the key information points from the story and, hence, increase the information score. Similarly, the mean length of utterance score does not reflect *quality* of expressive language as it measures only the length of the utterances. Two participants could score identically on mean length of utterance, but the complexity and variety of the language used in their utterances could be different (e.g., listing items within an utterance would increase the length of the utterance but not necessarily the complexity of the language used). Again, a strategy focused on producing a high volume of language is likely to inflate MLU scores.

The longitudinal design and population-based sample are strengths of this cohort study. However, the duration of the study, in which children have been followed up over many years, inevitably led to attrition of the study sample. The reduced sample of D/HH teenagers that provided spoken language data at the present assessment time point was similar to those that did not in terms of many key demographic characteristics, though there was some evidence of selective attrition of those participants who did not have English as a first language in the home, so caution should be exercised when generalizing these results to that population. The teenagers who provided spoken language data in this phase of the study showed higher T1 receptive language scores than those who did not. However, it is important to note that because this phase of the study collected spoken language data only from spoken language users, this meant that sign language users who were retained in this phase of the study were counted as nonparticipants for this examination of spoken language outcomes, despite some having provided receptive language data as children. This inflated the T1 receptive language difference between the participants and nonparticipants because these teenagers were more likely to have had low receptive language scores at T1; comparison of the overall retained and non-retained samples for this phase of the study which included these sign language users did not show higher receptive language skills in those who were retained.

Ideally, we would have been able to include both the spoken and sign language users within the same language analyses, but the lack of directly comparable standardized tests for speech and sign language users means it is difficult to make comparisons between the language skills of these two groups. As mentioned previously, the inclusion of only those participants who used spoken language reduced the sample size for these spoken language analyses; our work on reading comprehension outcomes in this cohort as teenagers (Pimperton et al. 2016) did include both speech and sign language users and found significant benefits of early confirmation to reading comprehension at the whole group level. The results in this article also do not address outcomes for those D/HH teenagers who have significant additional disabilities that preclude them from completing the language assessments. The effect of screening and early confirmation of PCHL on language outcomes for these individuals remains unquantified.

In summary, significant benefits of UNHS exposure on teenage spoken language outcomes were not demonstrated within the context of this study. Long-term significant benefits of early confirmation of PCHL to spoken language outcomes were only detectable for those D/HH teenagers who did not have CIs within this cohort and were not present for all language outcomes. High within-group variability, a sample size reduced by attrition, and a lack of sensitivity of some measures may have limited the power of this study to detect significant effects of early confirmation and of UNHS exposure; further examination of the effect of UNHS on spoken language outcomes when larger cohorts reach adolescence, including individual participant meta-analysis combining data across studies, would be valuable.

## ACKNOWLEDGMENTS

We thank the research assistants Eleanore Coulthard, Joanne Pickersgill, Lisa Shipway, and Zahra Taghizadeh; the audiologists Margaret Baldwin, Alyson Bumby, Adrian Dighe, Harpreet Nijar, David Reed, Joy Roberts, Sue Robinson, Salim Suleman, Rosbin Syed, and Huw Thomas; and the other medical and educational professionals who supported this study. We thank particularly the participating teenagers and their families. H. P. oversaw the conduct of the study, undertook statistical analysis, drafted the initial manuscript, and approved the final manuscript. J. K., M. M., J. S., and S. W. assisted in the design and supervision of the study, assisted with article preparation, and approved the final article. E. T. assisted in the supervision of the study and approved the final article. H. M. Y. undertook statistical analysis and approved the final manuscript. C. R. K. designed and supervised the study and the statistical analysis, assisted in manuscript preparation, and approved the final manuscript.
